# Imaging occult hemorrhage in polycystic kidney disease with red blood cell scintigraphy

**DOI:** 10.1007/s00259-026-07800-x

**Published:** 2026-02-13

**Authors:** Marco Bernhard, Cristina Popescu, Sabin G. Pop, Alexander W. Sauter, Florian Messmer, Erwin Grüter, Maria Wertli, Irene A. Burger

**Affiliations:** 1 Department of Internal Medicine, Kantonsspital Baden, Baden, Switzerland; 2 Department of Nuclear Medicine, Kantonsspital Baden, affiliated Hospital for Research and Teaching of the Faculty of Medicine of the University of Zurich, Baden, Switzerland; 3 Department of Radiology & Department of Nuclear Medicine, University Hospital Tuebingen, Tuebingen, Germany; 4 Department of Radiology, Kantonsspital Baden, affiliated Hospital for Research and Teaching of the Faculty of Medicine of the University of Zurich, Baden, Switzerland; 5 Department of Nephrology, Kantonsspital Baden, Baden, Switzerland; 6 Department of Nuclear Medicine, University Hospital Zurich, University of Zurich, Zurich, Switzerland; 7 Department of HEST, ETH Zurich, Zurich, Switzerland

Autosomal-dominant polycystic kidney disease (ADPKD) often presents with hematuria as in this case [1]. However, when conventional imaging (multiphase CT/MRI or angiography) is negative, localizing clinically relevant bleeding can be challenging [2]. In ADPKD patients the presence of cysts with residual blood accumulation, without active bleeding is a challenge for MRI/CT. We report the case of a man presenting with persistent gross hematuria requesting several blood transfusions, where in-vivo labelled [^99m^Tc]Tc-erythrocyte scintigraphy (RBC-scintigraphy) identified the bleeding site despite negative cystoscopy and CT angiography. RBC-scintigraphy has not been described for this clinical situation but is well established for occult gastrointestinal bleeding not seen on CT or MRI [3].

Standard protocol with 923 MBq [^99m^Tc]Tc-RBC was applied, with a dual-head SPECT/CT. Figure A shows, dynamic planar images with an increase in tracer accumulation after 50 min visualized on the right renal upper pole on posterior view (arrow). B) Subsequent quantitative coronal SPECT/CT after 60 min, confirming tracer within the renal pelvis (SUVmax 8.4, arrow). C) To rule out additional bleeding on the contralateral side, a second SPECT/CT was performed after 120 min, confirming an increased tracer accumulation (SUVmax 15.3, arrow), without evidence of bleeding on the left side. D) Embolization of the right kidney was successful, bleeding stopped thereafter, without the need for further blood transfusion.

When conventional imaging is inconclusive, the superior sensitivity of RBC scintigraphy with quantitative SPECT/CT for intermittent bleeding, can localize occult hemorrhage in ADPKD. Precise localization may support targeted embolization and avoid non-selective approaches when feasible.

**Figure:**
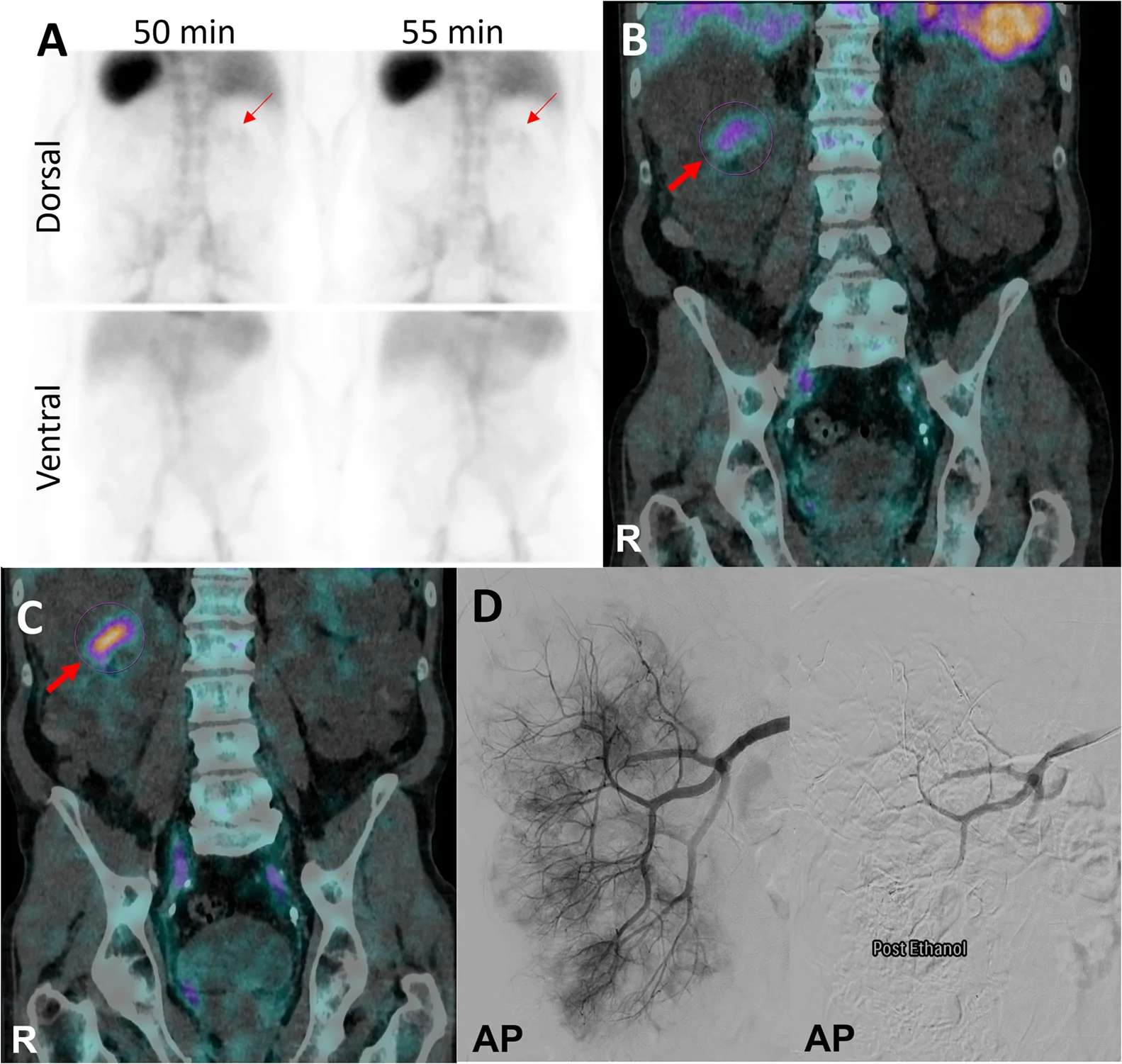

## Data Availability

The data analysed for this case study are available from the corresponding author on reasonable request.
